# Incidence and risk factors for delayed intracranial hemorrhage after mild brain injury in anticoagulated patients: a multicenter retrospective study

**DOI:** 10.1186/s13049-025-01337-y

**Published:** 2025-02-10

**Authors:** Nicolò Capsoni, Giovanni Carpani, Francesca Tarantino, Silvia Gheda, Jean Marc Cugnod, Sabrina Lanfranchi, Jhe Lee, Simone Lizza, Sara Marchesani, Enrica Meloni, Annalisa Rigamonti, Irene Serrai, Silvia Vergani, Elisa Ginevra Zuddio, Bruno Gherardo Zumbo, Daniele Privitera, Francesco Salinaro, Davide Bernasconi, Gianmarco Secco, Filippo Galbiati, Stefano Perlini, Michele Bombelli

**Affiliations:** 1 School of Medicine and Surgery, University of Milan-Bicocca, Milan, Italy; 2 Department of Emergency Medicine, ASST Grande Ospedale Metropolitano Niguarda, Milan, Italy; 3 Internal Medicine, Pio XI Hospital, ASST Brianza, Desio, Italy; 4 Department of Internal Medicine, University of Pavia, Pavia, Italy; 5 Emergency Medicine Unit, Fondazione IRCCS Policlinico San Matteo Pavia, Pavia, Italy; 6 Department of Emergency Medicine, Pio XI Hospital, ASST Brianza, Desio, Italy; 7 Department of Cardiovascular, Neural and Metabolic Sciences, Istituto Auxologico Italiano, IRCCS, San Luca Hospital, Milan, Italy; 8 Department of Clinical Research and Innovation, ASST Grande Ospedale Metropolitano Niguarda, Milan, Italy

**Keywords:** Brain injuries, Cerebral hemorrhage, Anticoagulants, Emergency department

## Abstract

**Background:**

Anticoagulated patients with mild traumatic brain injury (mTBI) and a negative cerebral CT on admission, commonly undergo a repeated CT scan after observation in the emergency department (ED) to detect delayed intracranial hemorrhage (ICH). However, the utility of this practice is controversial, with recent evidence suggesting that the risk of delayed ICH in these patients is low. This study aims to evaluate incidence, outcomes, and risk factors of delayed ICH in patients receiving direct oral anticoagulants (DOACs) or vitamin K antagonists (VKAs) presenting to the ED with mTBI.

**Methods:**

A multicenter, observational, retrospective cohort study was conducted in the EDs of three hospitals in Northern Italy, from January 2017 to December 2021. All consecutive adult patients on DOACs or VKAs therapy, admitted for a mTBI, who underwent a second CT scan after 12–24 h from a negative first one, were enrolled.

**Results:**

A total of 1596 anticoagulated patients were enrolled, 869 (54%) on DOACs and 727 (46%) on VKAs therapy. The median age was 84 [79–88] and 56% of patients were females. The incidence of delayed ICH was 1.8% (95% CI: 1.1-3.0%; 14/869 patients) for DOACs, and 2.6% (95% CI: 1.6–4.1%; 19/727 patients) for VKAs patients, with no cases requiring neurosurgical intervention. Vomiting after head injury and the onset of new symptoms during observation were associated with a higher risk of delayed bleeding (OR 4.8; 95% CI: 1.4–16.5, and OR 4.7; 95% CI 1.2–23.7, respectively). At a 30-day follow-up, 2% of patients had a new ED admission related to their previous mTBI, with no significant difference between the groups.

**Conclusions:**

Delayed ICH is uncommon among anticoagulated patients with mTBI and has minimal impact on their outcome. Routine performance of a second CT scan may be unnecessary and may be considered only in presence of high-risk clinical risk factors or signs of deterioration.

## Background

Traumatic brain injury (TBI) is a common cause of presentation to the Emergency Department (ED) with a high incidence rate, reaching 849 cases per 100,000 inhabitants annually in Europe [1]. The elderly are usually the most affected, with falls representing the most prevalent mechanism of trauma [1, 2, 3]. Mild traumatic brain injuries (mTBI), defined by a Glasgow Coma Scale (GCS) score of 14–15, account for at least three-quarters of TBI, and are typically associated with a favorable prognosis and a low risk of intracranial hemorrhage (ICH) [4, 5]. Factors that increase the risk of bleeding include advanced age, specific trauma dynamics, and antiplatelet or anticoagulant medications. Among these latter, direct oral anticoagulants (DOACs) have been demonstrated to be safer than vitamin K antagonists (VKAs) and associated with a lower risk of bleeding [6, 7, 8, 9, 10, 11]. 

Given the population aging and the spreading of anticoagulant use, the number of patients receiving anticoagulant therapy admitted to the ED for mTBI is increasing. Although all guidelines recommend performing a brain computed tomography (CT) in these patients at ED arrival, the subsequent management in the ED remains a topic of debate, with practices varying between centers. Several local ED protocols still include a follow-up brain CT after a 12-24-hour clinical observation, to rule out delayed post-traumatic ICH [12]. However, this practice remains controversial and has been recently questioned, with ongoing evidence showing that the routine use of a follow-up CT scan may be avoided both given the low incidence of delayed ICH in the absence of high-risk clinical risk factors or clinical deterioration, and given the increased radiation exposure and associated healthcare burden it is associated [3, 4, 5, 12, 13, 14, 15, 16, 17]. Reducing the number of repeated head CT scans and the length of observation in the ED of patients with mTBI on anticoagulation, may be beneficial both for better resource allocation in the EDs, which are often overcrowded and overwhelmed, and for patients, particularly the elderly and frail, for whom a prolonged ED stay may be associated with a higher risk of delirium, falls and short-term mortality [18, 19]. 

A better understanding of the incidence, prognosis and clinical features associated with a higher risk of delayed ICH is needed to improve the ED management of anticoagulated patients with mTBI.

Therefore, this study aims to evaluate the incidence, short-term outcomes and risk factors associated with delayed ICH in patients on DOACs and VKA therapy presenting to the ED with mTBI.

## Methods

### Study design and setting

A multi-center, observational, retrospective cohort study was conducted in the EDs of three hospitals in Lombardy, Italy: *(i)* ASST Grande Ospedale Metropolitano Niguarda, Milan (90’000 annual visits) *(ii)* IRCCS San Matteo, Pavia (80’000 annual visits) and *(iii)* Pio XI Hospital, Desio (65’000 annual visits). According to local protocols of the three EDs involved, all anticoagulated patients with mTBI received a first brain CT scan at arrival and a follow-up CT scan after 12 to 24 h of clinical observation. The enrollment covered a period of 5 years, from January 2017 to December 2021. The study was approved by the local ethical committee (Comitato Etico Territoriale Lombardia 3, ethical approval number 4839_22.05.2024_N). Owing to retrospective and de-identified data collection, the need for informed consent was waived.

### Participants

All consecutive patients admitted to the ED during the study period were enrolled if presenting the following inclusion criteria: (1) age ≥ 18 years, (2) mild traumatic brain injury defined as GCS score of 14–15 at ED presentation, (3) anticoagulation therapy including VKA and DOACs (Dabigatran, Rivaroxaban, Apixaban, Edoxaban), (4) a first negative brain CT scan performed on ED arrival within 24 h from head trauma, followed by a clinical observation in the ED and a follow-up brain CT performed between 12 and 24 h before hospital discharge. If patients presented to the ED more than 24 h after the mTBI, the first negative CT scan was considered definitive, and the patient was enrolled even without observation and CT scan repetition [20]. 

Exclusion criteria were: (1) ED presentation more than 48 h after TBI, (2) a GCS < 14 at arrival, (3) patients on ineffective anticoagulant therapy, defined as International Normalized Ratio (INR) < 1,7 in case of therapy with VKA therapy, or last anticoagulant intake more than 24 h before trauma in case of DOACs, (4) TBI following seizure, (5) a follow-up CT scan not performed before hospital discharge.

### Data search and collection

Data were collected retrospectively and extracted from the ED and hospital databases of each center. Patients were searched based on ED-coded and descriptive discharge diagnoses. To increase sensitivity, patients with the following diagnoses were searched and then screened and evaluated if eligible for enrollment: minor head trauma, concussive head trauma; non-concussive head trauma; head contusion; facial trauma; cerebral hemorrhage; subarachnoid hemorrhage; subdural hematoma; epidural hematoma; and descriptive diagnosis potentially associated to TBI (e.g. lacer contusion injury of the craniofacial district, fall to the ground, syncope, loss of consciousness, etc.).

On admission, patients’ demographic and clinical characteristics were collected including comorbidities, type and indication for anticoagulant therapy, intake of antiplatelet therapy, head injury characteristics (fall from orthostasis or higher energy impact), GCS score at ED presentation, syncope preceding the injury, evidence of trauma above the clavicles, signs of skull-base or other sites fractures, post-traumatic symptoms (including loss of consciousness, amnesia, seizures, headache, vomiting, confusion) and blood tests when available (platelets, INR, renal and hepatic function). Neurological examination variations and onset of new symptoms during ED observation (including state of consciousness alteration, headache, focal neurological deficit, nausea, dizziness, vomiting) together with time-lapse from TBI and the first and follow-up CT scan were recorded. Reversal of anticoagulation in the ED, ED disposal, discharge or admission to the hospital, temporary or permanent disposition to discontinue the anticoagulant therapy were collected. Readmission to the ED due to the previous TBI in the following 30 days was evaluated by consulting the hospital databases of each center.

### Study outcomes

The main outcome of the study was the incidence of delayed ICH in patients with mTBI on treatment with VKAs and DOACs. The delayed ICH was defined as the occurrence of subdural, epidural, subarachnoid, parenchymal hemorrhage, cerebral petechial hemorrhage, cerebral contusion, or subdural hygromas at the follow-up CT scan performed 12–24 h after the head trauma, following a first negative CT scan on ED admission.

The secondary outcomes were: the need for neurosurgical interventions (craniotomy, craniectomy, placement of a burr hole or subdural drain) and death due to intracerebral hemorrhage within 30 days from head trauma, and the evaluation of risk factors associated with delayed ICH. Thirty days patients’ follow-up outcomes were extracted from databases of the EDs involved in the study. For the evaluation of risk factors associated to delayed ICH demographic, comorbidities, concomitant antiplatelet therapy, clinical presentation, trauma dynamic, post traumatic symptoms and new symptoms onset during hospital observation were considered.

### Statistical analysis

Descriptive statistics were conducted on the entire sample and separately for subjects on VKAs and DOACs. Categorical/qualitative variables were expressed as absolute frequencies and percentages, continuous/quantitative variables as mean ± standard deviation or median and inter-quartile range, depending on whether or not the distribution was approximately normal. Differences between the two groups (VKAs and DOACs) were assessed with Chi-square and Fisher’s exact test or the T-test and Wilcoxon Mann Whitney test, as appropriate. Uni- and multivariable logistic models were implemented considering delayed ICH as outcome and various risk factors, including treatment (VKAs vs. DOACs), as covariates. Odds ratios (OR) with 95% confidence interval (CI) were estimated using the models.

Analyses were conducted using R software (version 4.3.2).

## Results

A total of 1596 anticoagulated subjects who presented to the ED with mTBI and performed a follow-up CT scan 12 to 24 h after the first negative one, were enrolled in the study. Of these, 901 (56,5%) were females, and the median age was 84 [79–88]. 869 subjects (54%) were on DOAC therapy, whereas 727 (46%) were on VKA therapy. Sixty-eight patients (8%) among the firsts and 46 (6%) among the seconds were on concomitant single or dual antiplatelet therapy. Demographics, comorbidities, and indications for anticoagulation therapy of the two patient groups (DOAC and VKA) are reported in Table 1. Most patients suffered a head injury falling from an orthostatic position (1543 patients, 97%) and presented with a GCS of 15 (1548 patients, 97%), with no differences among the DOACs and VKA groups. The first CT scan was performed after a median time of 3 [2–5] hours from the head injury, with no difference between the DOAC and VKA patients (*p* = 0.234). The follow-up imaging was performed after 23 [16–24] hours of observation, during which most of the patients reported no symptoms or changes in the clinical picture (1573 patients, 99%). Clinical presentation at arrival and symptoms reported during the ED observation time in the two study groups are reported in Table 2.

**Table 1:** Demographics, comorbidities and medications of the study population

	DOAC	VKA	
Total, *N (%)*	869 (100)	727 (100)	*P value*
**Demographics**			
Female, *N (%)*	502 (58)	399 (55)	0.268
Age, *median [IQR]*	84 [79–88]	83 [78–88]	0.415
**Comorbidities**			
Hypertension, *N (%)*	615 (71)	517 (71)	0.924
Diabetes, *N (%)*	142 (16)	157 (22)	0.009
Coronary artery disease, *N (%)*	219 (25)	265 (36)	< 0.001
Previous stroke or TIA, *N (%)*	150 (17)	93 (13)	0.016
Chronic renal failure, *N (%)*	105 (12)	158 (22)	< 0.001
Cirrhosis, *N (%)*	6 (1)	9 (1)	0.385
Coagulopathy, *N (%)*	14 (2)	31 (4)	0.002
Vascular disease, *N (%)*	148 (17)	130 (18)	0.704
**Anticoagulation drug**			
Apixaban, *N (%)*	319 (43)	-	-
Dabigatran, *N (%)*	150 (20)	-	-
Edoxaban, *N (%)*	104 (14)	-	-
Rivaroxaban, *N (%)*	171 (23)	-	-
Warfarin, *N (%)*	-	671 (93)	-
Acenocoumarol, *N (%)*	-	51 (7)	-
Reduced dosage of DOAC, *N (%)*	172 (20)	-	-
**Indication for anticoagulation**			
Atrial fibrillation, *N (%)*	728 (90)	517 (75)	< 0.001
Mechanical valve prothesis, *N (%)*	-	16 (2)	< 0.001
Venous thrombosis, *N (%)*	68 (8)	76 (11)	< 0.001
Other, *N (%)*	16 (2)	78 (11)	< 0.001
**Antiplatelets therapy**, *N (%)*	68 (8)	46 (6)	0.289
Dual Antiplatelet therapy, *N (%)*	3 (4)	1 (2)	0.746
Acetylsalicylic acid, *N (%)*	56 (82)	36 (78)	0.452
Clopidogrel, *N (%)*	14 (21)	10 (22)	0.452
Ticlopidine, *N (%)*	-	1 (2)	-

N: number, IQR: interquartile range, DOAC: Direct oral anticoagulant, TIA: transient ischemic attack, VKA: Vitamin K antagonist

**Table 2:** Trauma dynamic and clinical presentation at arrival and during ED observation

	DOAC	VKA	
Total, *N (%)*	869 (100)	727 (100)	*P value*
**Trauma dynamic**			
Fall from orthostatic position, *N (%)*	838 (96)	705 (97)	0.645
Higher energy dynamic, *N (%)*	31 (4)	22 (3)	0.645
**Clinical presentation at ED arrival**			
GCS score			
15, *N (%)*	840 (97)	708 (97)	0.487
14, *N (%)*	29 (3)	19 (3)	0.487
Altered neurological examination, *N (%)*	16 (2)	6 (1)	0.129
Post-traumatic LOC, *N (%)*	32 (4)	15 (2)	0.079
Post-traumatic amnesia, *N (%)*	56 (6)	31 (4)	0.072
Post-traumatic seizures, *N (%)*	0 (0)	4 (1)	0.092
Post-traumatic headache, *N (%)*	18 (2)	15 (2)	1
Post-traumatic vomiting, *N (%)*	24 (3)	11 (1)	0.127
Post-traumatic confusion, *N (%)*	27 (3)	21 (3)	0.915
Post-syncopal head trauma, *N (%)*	122 (14)	76 (10)	0.037
Signs of skull base fracture, *N (%)*	4 (0)	2 (0)	0.848
Signs of supraclavicular trauma, *N (%)*	291 (33)	349 (48)	< 0.001
Presence of other fractures, *N (%)*	128 (15)	95 (13)	0.378
**Blood tests**			
Platelets, cell/L^− 1^, *median [IQR]*	202 [170–248]	194 [156–233]	< 0.001
INR, *median [IQR]*	1.17 [1.1–1.3]	2.32 [1.9–2.9]	< 0.001
aPTT ratio, *median [IQR]*	28.5 [22.9–34.9]	36.7 [23.8–44.8]	< 0.001
Creatinine, mg/dL, *median [IQR]*	1.02 [0.81–1.38]	1.05 [0.83–1.46]	0.108
Alanine aminotransferase, *median [IQR]*	16 [12–23]	17 [12–22]	0.772
Total bilirubin, mg/dL, *median [IQR]*	0.59 [0.4–0.83]	0.56 [0.4–0.81]	0.612
**Symptoms and signs during ED observation**			
None, *N (%)*	858 (99)	715 (98)	0.199
New symptoms and signs onset, N (%)	13 (1)	12 (2)	0.199
State of consciousness alteration, *N (%)*	4 (0)	3 (0)	0.199
Headache, *N (%)*	3 (0)	4 (1)	0.199
Focal neurological deficit, *N (%)*	0 (0)	2 (0)	0.199
Nausea, *N (%)*	3 (0)	0 (0)	0.199
Dizziness, *N (%)*	0 (0)	2 (0)	0.199
Vomiting, *N (%)*	3 (0)	1 (0)	0.199

N: number, IQR: interquartile range, aPTT: activated partial thromboplastin time, DOAC: Direct oral anticoagulant, ED: emergency department, GCS: Glasgow Coma Scale, INR: International Normalized Ratio, LOC: loss of consciousness, VKA: Vitamin K antagonist

The incidence of delayed ICH detected at the follow-up CT scan was 1.8% (95% CI: 1.1-3.0%; 14/869 patients) for patients on DOACs therapy, and 2.6% (95% CI: 1.6–4.1%; 19/727 patients) among those on VKAs therapy. None of these intracerebral bleedings underwent neurosurgical intervention (Table 3 for details). No differences in terms of delayed ICH were observed among the four DOACs (Dabigatran 2.9%, Rivaroxaban 1.2%, Apixaban 1.2%, Edoxaban 2.6%, *p* = 0.401), neither between the two VKAs (Coumadin 2.5%, Sintrom 3.9%, *p* = 0.549). One of the 14 patients on DOAC with delayed ICH received prothrombin complex concentrate (PCC), whereas 7 of those on VKA received vitamin K and among them 2 received PCC.

**Table 3:** Study outcomes

	DOAC	VKA	
Total, *N (%)*	869 (100)	727 (100)	*P value*
**At first ED access**			
Delayed ICH at follow-up CT scan, *N (%)*	14 (1.8)	19 (2.6)	0.221
Delayed ICH characteristics:			
Parenchymal hematoma, *N (%)*	0 (0)	3 (16)	-
Subdural hematoma, *N (%)*	3 (21)	6 (31)	-
Subarachnoid hemorrhage, *N (%)*	6 (43)	7 (37)	-
Petechial cerebral hemorrhage, *N (%)*	4 (28)	1 (5)	-
Subdural hygroma, *N (%)*	2 (14)	2 (10)	-
Neurosurgical intervention, *N (%)*	0 (0)	0 (0)	-
ED discharge, *N (%)*	803 (92)	668 (92)	0.77
Hospitalization, *N (%)*	66 (8)	59 (8)	0.77
Disposition to AC discontinuation, *N (%)*	353 (41)	228 (31)	< 0.001
Number of days, *N (%)*:			
1	291 (86)	160 (73)	< 0.001
2	18 (5)	32 (14)	< 0.001
3	11 (3)	10 (4)	< 0.001
4	1 (0)	6 (3)	< 0.001
5	6 (2)	2 (1)	< 0.001
> 30	5 (1)	6 (3)	< 0.001
**Within 30 days follow-up**			
Readmission to the ED, *N (%)*	17 (2)	8 (1)	0.242
Neurosurgical intervention	0 (0)	0 (0)	-
In-hospital mortality	2 (0)	1 (0)	1

N: number, AC: anticoagulation, DOAC: Direct oral anticoagulant, ED: emergency department, ICH: intracranial hemorrhage, VKA: Vitamin K antagonist

After adjusting for possible confounders with a multivariable model considering the overall population, the occurrence of vomiting following head injury and the onset of new symptoms during the observation in the ED (including state of consciousness alteration, headache, focal neurological deficit, nausea, dizziness and vomiting) were associated with an increased risk of delayed bleeding at the follow-up CT scan (OR 4.8; 95% CI: 1.4–16.5, and OR 4.7; 95% CI 1.2–23.7, respectively). Univariable and multivariable models are reported in Table 4. Evaluating the DOAC group individually, no differences between reduced and normal dosages were shown in terms of risk of delayed bleeding (OR 0.3; 95% CI: 0.4–2.4). Individually assessing patients on VKA therapy revealed no increased risk of delayed bleeding with higher INR values (OR 1.04; 95% CI: 0.4–2.4, for every decimal unit of INR increase).

**Table 4:** Logistic regression analysis to assess the relationship between delayed ICH and demographic and clinical variables

	Univariable models		Multivariable model	
	OR (95% CI)	*P-value*	OR (95% CI)	*P-value*
**Demographics**				
Male vs. Female,	1.8 [0.9–3.6]	0.105		
Age	1.0 [1.0-1.1]	0.140		
**Comorbidities**				
Hypertension	0.9 [0.4-2.0]	0.875		
Diabetes	1,0 [0.4–2.4]	0.934		
Coronary artery disease	0.5 [0.2–1.2]	0.132		
Previous stroke or TIA	0.8 [0.3–2.2]	0.618		
Chronic renal failure	1.1 [0.5–2.8]	0.790		
Cirrhosis	-*	-		
Coagulopathy	1.1 [0.1–8.1]	0.941		
Vascular disease	0.8 [0.3–2.1]	0.729		
**Anticoagulation drug**				
VKAs vs. DOACs	1.6 [0.8–3.3]	0.165	1.8 [0.9;3.6]	0.122
**Antiplatelets therapy**	0.4 [0.1-3.0]	0.370		
**Dual Antiplatelet therapy**	-*	-		
Major trauma dynamic	1.9 [0.5–8.2]	0.383	1.8 [0.4;8.2]	0.421
GCS score	-*	-		
Altered neurological examination	2.3 [0.3–17.6]	0.424		
Post-traumatic LOC	2.2 [0.5–9.4]	0.297	2.0 [0.5;9.2]	0.361
Post-traumatic amnesia	1.8 [0.6–5.9]	0.358		
Post-traumatic seizures	-*	-		
Post-traumatic headache	1.5 [0.2–11.3]	0.696		
Post-traumatic vomiting	4.8 [1.4–16.5]	0.013	5.0 [1.4;17.9]	0.013
Post-traumatic confusion	2.1 [0.5–9.2]	0.311		
Post-syncopal head trauma	0.5 [0.1–1.9]	0.276		
Signs of skull base fracture	-*	-		
Signs of supraclavicular trauma	1.6 [0.8–3.2]	0.180		
Presence of other fractures	1.4 [0.6–3.4]	0.483		
**Blood tests**				
Platelet	1.0 [1.0-1.1]	0.958		
INR	1.2 [0.8;1.7]	0.410		
aPTT ratio	1.0 [1.0-1.1]	0.741		
Creatinine	1.1 [0.6–1.9]	0.701		
Alanine aminotransferase	1.0 [1.0–1.0]	0.965		
Total bilirubin	1.8 [0.8–3.9]	0.152		
**Neurological changes during ED observation**	2.2 [0.3;16.7]	0.450		
**New symptoms onset during ED observation**	4.7 [1.1–21.1]	0.041	5.3 [1.2;23.7]	0.031

N: number, AC: anticoagulation, DOAC: Direct oral anticoagulant, ED: emergency department, GCS: Glasgow Coma Scale, ICH: intracranial hemorrhage, LOC: loss of consciousness, OR: odds ratio, TIA: transient ischemic attack, VKA: Vitamin K antagonist

* it was not possible to estimate the OR because all 33 events were observed in one level of the covariate (i.e. no cirrhosis, no dual Antiplatelet therapy, GCS = 15, no post-traumatic seizures and no signs of skull base fracture)

At a 30-day follow-up, considering the overall population, 25 (2%) patients underwent a new ED admission due to a complaint referable to the previous head injury, with no difference between the two groups (0.242). None of these required a neurosurgical intervention. The overall 30-day mortality was 0.2% (3/1596 patients, including 2 patients in the DOAC group and 1 patient in the VKA group), but none of these deaths were related to post-traumatic ICH (Table 3).

## Discussion

The study aimed to evaluate the incidence, short-term outcomes and risk factors associated with delayed ICH in a large sample of consecutive patients on DOACs and VKAs therapy, presenting for mTBI to the ED of three hospitals in northern Italy. The main findings of the study are: *(i)* the incidence of delayed ICH at a follow-up CT scan performed 12–24 h after a first negative CT scan performed at arrival is low both in patients receiving DOACs and VKAs, *(ii)* none of the patients with delayed ICH required neurosurgical intervention or died at 30 days follow-up due to ICH, *(iii)* the occurrence of vomiting following head injury and the onset of new symptoms during the observation in the ED was associated with an increased risk of delayed ICH.

To date, the routine repetition of a brain CT scan after an initial negative one after a mTBI is still a common practice in patients receiving DOACs or VKAs, even if poorly supported by literature. Recently, evidence that the routine use of a follow-up CT scan may be unnecessary is growing, showing a low incidence of delayed traumatic ICH in the absence of high-risk clinical risk factors or clinical deterioration. Reducing the observation time in the ED and the number of unnecessary brain CT scans performed, may allow for reduction of radiation exposure, healthcare burden and ED overcrowding, which is associated to worse patients’ outcomes [18, 19]. 

A low incidence of delayed ICH was found for both patients on DOACs (1.8% 95%CI: 1.1-3.0%) and patients on VKAs (2.6% 95%CI: 1.6–4.1%). Furthermore, none of the patients diagnosed with an ICH at the second CT underwent neurosurgical intervention, and neither died due to cerebral bleeding. These results confirm the findings of previous studies which reported an incidence of delayed ICH ranging from 0,5 to 4% and a generally favorable short- and middle-term prognosis in both DOACs and VKAs patients, with minimal instances of the need for neurosurgical intervention [5, 13, 14, 15, 16, 21, 22]. While patients on VKA therapy are generally at increased risk of early intracranial bleeding following TBI compared to patients on DOACs, no such difference has been demonstrated when only delayed ICHs are considered. In line with previous literature, no significant difference between the two groups was observed in our study [11]. 

To better identify patients with mTBI deserving deeper monitoring in the ED, including prolonged observation and eventually a repeated CT scan, previous studies have sought to identify potential risk factors for delayed intracranial hemorrhage (ICH) among patient characteristics, trauma dynamics, and post-traumatic symptoms. Previously, Turcato et al., reported that a high-energy impact, post-traumatic loss of consciousness, or post-traumatic amnesia, a GCS lower than 15, and evidence of supraclavicular injury at ED arrival, are associated with a higher risk of delayed ICH [5, 14]. Although these risk factors were not confirmed in our cohort in multivariate analyses, post-traumatic vomiting and the onset of new neurological symptoms (including state of consciousness alteration, headache, focal neurological deficit, nausea, dizziness and vomiting) were shown to be associated with a higher risk of delayed ICH (OR 4.8; 95% CI: 1.4–16.5, and OR 4.7; 95% CI 1.2–23.7, respectively). Even if these findings were not exactly consistent with those previously reported in the literature, they confirm and highlight the importance of evaluating post-concussive symptoms when stratifying the patient’s severity. Furthermore, they stress the importance of conducting clinical observation after the head trauma, whether in the ED or after discharge, to monitor neurological deterioration and the onset of new alarming symptoms to identify patients who warrant a repeated brain CT scan.

These results should be interpreted with caution, considering several limitations of the study. Firstly, the retrospective design may have resulted in some inaccuracy in the enrolment process and an increase in the number of missing data. On the other hand, it is also important to note that the patient’s search in hospital databases was conducted using both coded and free-text ED discharge diagnoses. This approach was taken to ensure a broad and sensitive search, to minimize selection bias and inaccuracy in the estimation of delayed ICH incidence. Furthermore, it should be emphasized that the retrospective enrolment is more likely to have resulted in an overestimation of incidence rather than an underestimation. This is because patients with ICH typically have a well-documented diagnosis and are rarely lost during database searches, in contrast to patients with mTBI without bleeding complications. Secondly, the 30-day follow-up was conducted by evaluating only readmissions to the same hospital as the initial ED arrival, thus limiting the reliability of 30-day outcomes data. Thirdly, the identification of risk factors was constrained by two factors: the number of missing data resulting from the retrospective data collection methodology, and the low incidence of delayed ICH. To address this issue, the two patient groups (DOACs and VKAs) were merged, which may have partially compromised the reliability of the results within each group. Lastly, the enrolment period encompassed the course of the SARS-CoV-2 pandemic, during which a slight reduction and selection of patients admitted to the emergency department (ED) were observed, together with reduced adherence to ED protocols due to ED overcrowding and contagion risk.

Despite the aforementioned limitations, the extensive study population, which is one of the largest cohorts to examine the incidence of delayed intracerebral hemorrhage in anticoagulated patients, and the multicenter recruitment strategy, may enhance the generalizability and clinical relevance of the findings. Given the low frequency of delayed ICH in anticoagulated patients, it may be advisable to consider the potential benefits and safety of eliminating the routine follow-up CT scan at 12–24 h for all patients with mild traumatic brain injury (mTBI) who are anticoagulated. The assessment of risk factors, such as the presence of post-concussive symptoms and the observation of clinical signs following mTBI is of paramount importance to identify patients who require a deeper monitoring and a follow-up CT scan.

## Conclusion

Delayed intracerebral hemorrhage is an uncommon occurrence among anticoagulated patients with mTBI and it has a minimal impact on their prognosis. It may be advisable to refrain from the routine use of a second CT scan, and to consider it only in cases where there are high-risk clinical risk factors or signs of deterioration. Further studies focusing on risk stratification may assist clinicians in identifying patients who require closer monitoring and more intensive management following mTBI, thereby enabling more effective resource allocation.

## Data Availability

No datasets were generated or analysed during the current study.

## Abbreviations

CI: Confidence interval
CT: Computed tomography
DOACs: Direct oral anticoagulants
ED: Emergency department
GCS: Glasgow Coma Scale
ICH: Intracranial hemorrhage
INR: International Normalized Ratio
IQR: Interquartile range
mTBI: Mild traumatic brain injuries
TBI: Traumatic brain injury
VKAs: Vitamin K antagonists
